# A distinct molecular profile associated with mucinous epithelial ovarian cancer

**DOI:** 10.1038/sj.bjc.6603003

**Published:** 2006-02-28

**Authors:** V A Heinzelmann-Schwarz, M Gardiner-Garden, S M Henshall, J P Scurry, R A Scolyer, A N Smith, A Bali, P Vanden Bergh, S Baron-Hay, C Scott, D Fink, N F Hacker, R L Sutherland, P M O'Brien

**Affiliations:** 1 Cancer Research Program, Garvan Institute of Medical Research, Darlinghurst, NSW 2010, Australia; 2 Division of Gynecology, University Hospital Zurich, Switzerland; 3 South Eastern Area Laboratory Service, Prince of Wales Hospital, Randwick, NSW 2031, Australia; 4 Department of Anatomical Pathology, Royal Prince Alfred Hospital, Camperdown, NSW 2050, Australia; 5 Kolling Institute of Medical Research, Royal North Shore Hospital, St Leonards, NSW 2065, Australia; 6 Gynaecological Cancer Centre, Royal Hospital for Women, Randwick, NSW 2031, Australia

**Keywords:** ovarian cancer, mucinous, microarray, immunohistochemistry, diagnosis

## Abstract

Mucinous epithelial ovarian cancers (MOC) are clinically and morphologically distinct from the other histological subtypes of ovarian cancer. To determine the genetic basis of MOC and to identify potential tumour markers, gene expression profiling of 49 primary ovarian cancers of different histological subtypes was performed using a customised oligonucleotide microarray containing >59 000 probesets. The results show that MOC express a genetic profile that both differs and overlaps with other subtypes of epithelial ovarian cancer. Concordant with its histological phenotype, MOC express genes characteristic of mucinous carcinomas of varying epithelial origin, including intestinal carcinomas. Differences in gene expression between MOC and other histological subtypes of ovarian cancer were confirmed by RT–PCR and/or immunohistochemistry. In particular, galectin 4 (LGALS4) was highly and specifically expressed in MOC, but expressed at lower levels in benign mucinous cysts and borderline (atypical proliferative) tumours, supporting a malignant progression model of MOC. Hence LGALS4 may have application as an early and differential diagnostic marker of MOC.

Carcinomas arising from the epithelial cells of the ovary are the fifth most common malignancy in women and the leading cause of death from gynaecological cancers. Epithelial ovarian cancers comprise a group of related but distinct carcinomas that likely arise from a common epithelial cell type but develop via differentiation pathways and differ in their clinical presentation and aetiology. They are currently classified into different histological subtypes (including serous, endometrioid, mucinous and clear cell) based on their morphological resemblance to normal epithelia in the gynaecological and intestinal tracts; however the genetic basis underlying their divergence is poorly understood.

The majority of mucinous ovarian cancers (MOC) are diagnosed at an early stage, either as borderline (atypical proliferative) tumours or low-grade carcinomas, and have an excellent prognosis (Sherman *et al*, 2004). Although less common, advanced MOC is associated with a very poor survival that surpasses the poor prognosis for women with advanced stage serous ovarian cancer (Sherman *et al*, 2004). Accumulating pathological and epidemiological evidence supports a progression model of MOC, from benign cysts to borderline tumours to invasive adenocarcinoma (Feeley and Wells, 2001; Shih and Kurman, 2004). However, it can be difficult to identify invasion which may only be focally present and thus such tumours, particularly those of large size, must be extensively sampled for accurate diagnosis (Riopel *et al*, 1999; Lee and Young, 2003; Seidman *et al*, 2003; Ronnett *et al*, 2004). Moreover, it can be very difficult to differentiate primary MOC from secondary mucinous carcinomas from other sites, in particular the gastrointestinal tract (Hart, 2005). Indeed it is thought that many carcinomas diagnosed as primary mucinous epithelial ovarian cancer are likely metastatic disease, and that the true frequency of mucinous carcinoma arising in the ovary is <3% of all ovarian carcinomas (Gilks, 2004; Seidman *et al*, 2004). Hence early diagnosis and accurate classification of MOC, including the ability to identify patients who are likely to progress to invasive disease, is critical to patient prognosis and treatment (Hart, 2005).

The molecular basis of MOC, including the genetic events that initiate the development of disease and those leading to malignant progression, are largely unknown. One genetic abnormality characteristic of MOC is a high frequency of mutations in *KRAS*, thought to occur early in the development of MOC (Feeley and Wells, 2001). Unlike serous ovarian carcinomas, mutations in *p53* are rarely observed in MOC (Shih and Kurman, 2004). In our laboratory, we have successfully applied transcript profiling of whole tissue as a screening tool to determine molecular changes underlying cancer, which has led to the identification of several potential markers for prostate, ovarian and pancreatic cancer (Henshall *et al*, 2003a, 2003b; Heinzelmann-Schwarz *et al*, 2004; Segara *et al*, 2005). In the current study, we have determined the gene expression profiles of mucinous borderline tumours and MOC using oligonucleotide microarrays representing over 90% of the expressed human genome. By comparing the results to transcript profiles of the other histological subtypes of ovarian cancer, we aimed to determine the molecular basis of mucinous ovarian tumours and to identify potential tumour markers. Following validation of the transcript profiling results using RT–PCR analysis on ovarian cancer extracts of varying histological subtypes, we determined the protein expression of one such candidate tumour marker, galectin 4 (*LGALS4*), in primary ovarian tissue (normal surface epithelium, benign mucinous cysts, mucinous borderline tumours and ovarian carcinomas) using high-throughput immunohistochemistry based on tissue microarrays.

## MATERIALS AND METHODS

### Tissue and clinicopathological data

Tissue specimens (fresh/frozen and formalin-fixed paraffin-embedded samples) collected from patients undergoing primary laparotomy at the Gynaecological Cancer Centre, Royal Hospital for Women, Sydney, and the Royal North Shore Hospital, Sydney, between 1990 and 2003 were included in this study following informed consent and approval by the appropriate hospital research ethics committee. The histological classification at diagnosis was independently confirmed by a gynaecological pathologist for all tissue specimens before inclusion in the study. Normal ovaries were obtained from patients undergoing surgery for benign gynaecological conditions or unrelated malignancies. Patients exhibiting clinical, morphological or microscopic features suggesting metastatic mucinous ovarian carcinoma rather than primary MOC, including concurrent gastrointestinal carcinomas, the presence of *Pseudomyxoma peritonei/ovarii*, bilateral disease, Krukenberg tumours, and advanced stage borderline tumours (Lee and Young, 2003; Seidman *et al*, 2003; Hart, 2005), were excluded from the study. The clinical and pathological details of the tissue cohort used in this study are shown in Table 1.

### Molecular profiling and data analysis

Transcript profiling was performed as previously described (Heinzelmann-Schwarz *et al*, 2004) using the Eos Hu03, a customised Affymetrix GeneChip® oligonucleotide microarray containing over 59 000 probesets for the interrogation of approximately 46 000 unique sequences (Eos Biotechnology/Protein Design Labs, Fremont, CA, USA; Platzer *et al*, 2002) using total RNA extracted from three MOC (stage I), four mucinous borderline tumours, eight endometrioid ovarian cancers, 31 serous ovarian cancers, three serous borderline tumours, and four normal ovaries. Only those tumour samples containing >75% of borderline or invasive cancer were used for transcript profiling. Following normalisation as described (Henshall *et al*, 2003a), data was log-transformed before further analysis. In addition, prior to hierarchical clustering or principal components analysis, the data were scaled to ensure that each gene exhibited the same mean and variance. Principal components analysis was used to provide a visual demonstration of the variation in gene expression of the top ranked between ovarian cancer histological subtypes using the Stats package in R (http://www.r-project.org; Smyth, 2004). Hierarchical clustering of genes and samples was performed using an euclidean distance metric with average linkage (Spotfire DecisionSite 8.0).

A penalised *t*-test (Lönnstedt and Speed, 2002; Smyth, 2004) was used to identify genes differentially regulated between MOC and other subtypes of ovarian cancer. *P*-values were adjusted for multiple testing using the Benjamini-Yekutieli method (Benjamini and Yekutieli, 2001). Genes with an adjusted *P*-value <0.01 can be interpreted as having a false discovery rate of 1%. Genes were assigned to functional categories (molecular function, biochemical process, cellular localisation, chromosome) using Gene Ontology (http://vortex.cs.wayne.edu/projects.htm; Draghici *et al*, 2003) and GenMAPP (www.genmapp.org) analysis (Dahlquist *et al*, 2002).

### RT–PCR

RNA (2 *μ*g) was treated with DNAse then reverse-transcribed using the Reverse Transcription System (Promega, Australia) according to the manufacturer's instructions. Semi-quantitative RT–PCR was performed by the amplification of selected gene transcripts using 2 *μ*l of the resulting cDNA in a 25 *μ*l reaction volume incorporating 200 *μ*M of dNTPs (Roche, Australia), 2.5 mM MgCl_2_, 1.5 U of Amplitaq Gold (Promega), and 1 *μ*M of each oligonucleotide pair. Oligonucleotide primers and PCR product size for each gene were as follows: LGALS4: forward 5′ GCTCAACGTGGGAATGTCTGTTTAC, reverse 5′ TTGTAGTGCTCAGCCAGGACTATG (260 bp); MGC32871: TGAGTCACGGACTTGCAG, TTCGCCACAAACAGTATCA (260 bp); MUCDHL: AATGTGGAACCCAGCCACA, CACGTTTCCCCTAAAGATGCT (270 bp); CDH17: CTTCACTCCCTGTGTCTTCTTATGC, CCTGTCCAAGGCTCTGTTGTAATAC (240 bp); MEP1A: GGTTTCATCTCCCACCAAATGC, AGGTACGGCTTCCTCTAACATGG (220 bp); MUC13: GCATTTGGCTACAGTGGACTCG, CTTAGGAAAGACGCTCCCTTCTG (240 bp); FABP1: GAGCCAGGAAAACTTTGAAGCC, TGGTGATTATGTCGCCGTTGAG (300 bp); C19orf21: CCAACGCCAGATGAGAACT, CGTTCTTGTGACGGGTC (260 bp); MUC2: CGGTAAAACGACCCCACACAAG, CATCAAAGCCAGGAGCGTAGTTC (400 bp); TFF1: TGGAGAACAAGGTGATCTGCG, AAACAGCAGCCCTTATTTGCAC (160 bp); REGIV: TATCAGAGAAGCCAGCCGATATG, TTGCACAGGAAGTGTTGGCG (200 bp). Amplification of GAPDH (GTCCACTGGCGTGTTCACCA, GTGGCAGTGATGGCATGGAC, 260 bp) was used as a control. Cycling commenced with a 12 min heat activation at 95°C, followed by 24 cycles of strand denaturation at 95°C for 30 s, annealing at 55–60°C for 30 s, and extension at 72°C for 1 min. A final extension time of 7 min followed the last cycle. Products were separated on a 2% agarose gel in Tris-Acetate-EDTA buffer and visualised using ethidium bromide staining.

### Immunohistochemistry

Protein expression of LGALS4 was determined in a cohort of fixed tissue from 124 patients with ovarian cancer (comprising 10 MOC (independent of the samples that were transcript profiled), 55 serous ovarian cancers, 22 endometrioid ovarian cancers, eight clear cell ovarian cancers, and 29 mucinous borderline tumours; Table 1). In addition, eight benign mucinous cysts and 14 normal ovaries, some of which contained inclusion cysts (sites of enclosed metaplastic epithelium proposed as a precursor lesion for some ovarian carcinomas; Feeley and Wells, 2001), were used for immunohistochemistry. All tissues were incorporated into tissue microarrays following pathological review, with each patient represented by two to five tissue cores.

Tissue sections (4 *μ*m) were dewaxed and rehydrated according to standard protocols, and endogenous peroxidase blocked using 3% H_2_O_2_. Sections were treated with proteinase K to facilitate antigen retrieval, followed by incubation for 1 h with goat anti-galectin 4, 1:100 (sc19286, Santa Cruz Biotechnology, CA, USA). Bound antibody was detected using the LSAB+Kit/HRP and DAB+(diaminobenzidine) (DAKO Cytomation) and hematoxylin counterstaining. A negative control omitted the primary antibody, and a positive (small bowel) and negative (testis, skeletal muscle) control tissue was included. Scoring was independently assessed by two observers trained in gynaecological pathology and discrepancies resolved by consensus. All cells within each core were counted and the percentage of cells staining for each core determined. The average percentage staining of multiple cores was calculated for each patient. Box and whisker plots showing staining distributions (median and 25th–75th percentile range) were produced using the Base library in R (http://www.r-project.org). The median is marked as a horizontal line between the box edges, which represent the 25th and 75th percentile values. The length of the whiskers is 1.5 times the interquartile range and values outside this range are marked as circles. Differences in protein expression were determined using the Mann–Whitney *U*-test, and correlations between gene expression and clinicopathological parameters were analysed using Fisher's exact test. A *P*-value of ⩽0.05 was required for significance. All statistical analyses were performed using Statview 4.5 software (Abacus Systems, Berkeley, CA, USA).

## RESULTS

### MOC exhibit a gene expression profile distinct from other ovarian cancers

**Figure 1:**
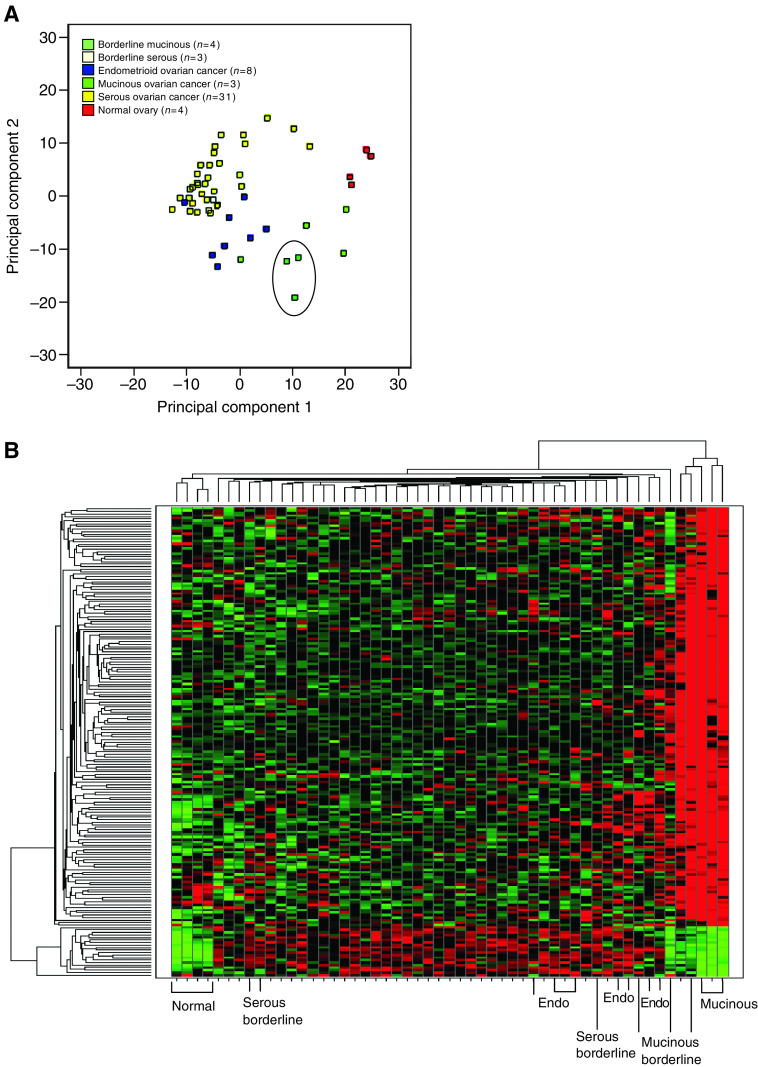
(**A**) Principal components analysis based on 500 genes with the most variable signal intensities (based on variance) separates the histological subtypes of EOC. MOC (*n*=3) are circled; (**B**) Hierarchical clustering and heat map of differentially expressed genes (*n*=167 upregulated and *n*=18 down-regulated) in MOC compared to serous and endometrioid ovarian cancers. Clustering was performed on all transcript profiled samples (*n*=3 MOC; *n*=4 mucinous borderline tumours; *n*=8 endometrioid ovarian cancers (endo); *n*=3 serous borderline tumours; *n*=31 serous ovarian cancers (unlabelled columns); and four normal ovaries) as described in the Materials and Methods. Expression levels are colour coded with red, green and black corresponding to an increase, a decrease, and no change in gene expression, respectively.

Principal components analysis on the top 500 most variable genes identified by transcript profiling showed that MOC can be clearly distinguished from the other subtypes of ovarian cancer by their expression profile, and cluster more closely to endometrioid ovarian cancer than to serous carcinomas (Figure 1A), as previously observed (Schwartz *et al*, 2002; Hart, 2005). Using a penalised *t*-statistic, we identified 167 probesets with higher expression in MOC compared to serous and endometrioid ovarian cancers (*P*-value adjusted for multiple testing <0.01) (Table 2 and Supplementary Data), and 18 probesets whose expression was lower in MOC compared to the other cancers (Supplementary Data). Hierarchical clustering illustrated that these genes can clearly separate MOC from the other subtypes of ovarian cancer, and shows that in most cases mucinous borderline tumours cluster closely with MOC (Figure 1B). Genes identified as having low expression in MOC compared to the other subtypes had similar expression levels in normal (whole) ovaries (Figure 1B), and their identification here may reflect their high expression in serous/endometrioid ovarian cancers rather than reduced expression in MOC.

In all, 40 genes with higher expression in MOC compared to normal ovaries were identified (Table 3). As the normal ovaries were not microdissected before RNA extraction and profiling and therefore contain a high proportion of stromal tissue compared to epithelial cells, these genes likely reflect epithelial-specific genes expressed in MOC. Nonetheless, the majority of these genes are common to all subtypes of ovarian cancer, and several have been previously implicated in its pathogenesis, including *TACSTD1* (Ep-CAM), *CDH1* (E-cadherin), *KLF5* (Kruppel-like factor 5) and *ERB-B3* (Darai *et al*, 1997; Balzar *et al*, 1999; Maihle *et al*, 2002; Heinzelmann-Schwarz *et al*, 2004). Combining the two analyses, we identified that 13 of these 40 genes overlap with those that are upregulated in MOC compared to the other subtypes of ovarian cancer (highlighted in Table 3). Only four genes were identified as down-regulated in MOC compared to normal ovaries (adjusted *P*<0.01) (Table 3), all of which are also reduced in the other subtypes of ovarian cancer.

We next clustered the upregulated genes in MOC compared to the other subtypes by their chromosomal location and identified several genomic regions that appeared to be over-represented in MOC, including 3p21.3 (*VILL*, *MST1R*, *SLC26A6*, *GLYCTK*, *FLJ20209*), 7q22 (*MUC3B*, *ACHE*, *MUC17*, *CLDN15*, *LOC55971*), 11p15 (*USH1C*, *MUCDHL*, *MUC2*, *SLC22A18*), 11q13 (*STATD10*, *PLCB3*, *MOGAT2*), 11q24 (*CTXL*, *KIAA1201*, *LOC120224*, *RICS*), 15q14-15 (*PPP1R14D*, *ITPKA*, *CKMT1*, *NMES1*), 19p13.3 (*FUT3*, *FLEKHJ1*, *C19orf21*, *LOC284422*, *GNA11*), 19q13.1-13.4 (*CYP2S1*, *FXYD3*, *LGALS4*, *CEACAM5*, *CEACAM6*, *FLJ20200*. *PTPRH*), and 20q13 (*HNF4A*, *BCAS1*, *PTK6*). Chromosomes 11q24, 19q13.2, and 20q13 have been previously associated with a high frequency of loss of heterozygosity in MOC (Feltmate *et al*, 2005). Together these data suggest chromosomal amplification affecting these genomic loci in MOC. Moreover, both 3p21.3 (*MST1R*) and 20q13 (*PTK6*) contain putative oncogenes (Barker *et al*, 1997; Hess *et al*, 2003; Maggiora *et al*, 2003; Wang *et al*, 2003), which are frequently located in regions of genomic amplification in cancer.

**Figure 2:**
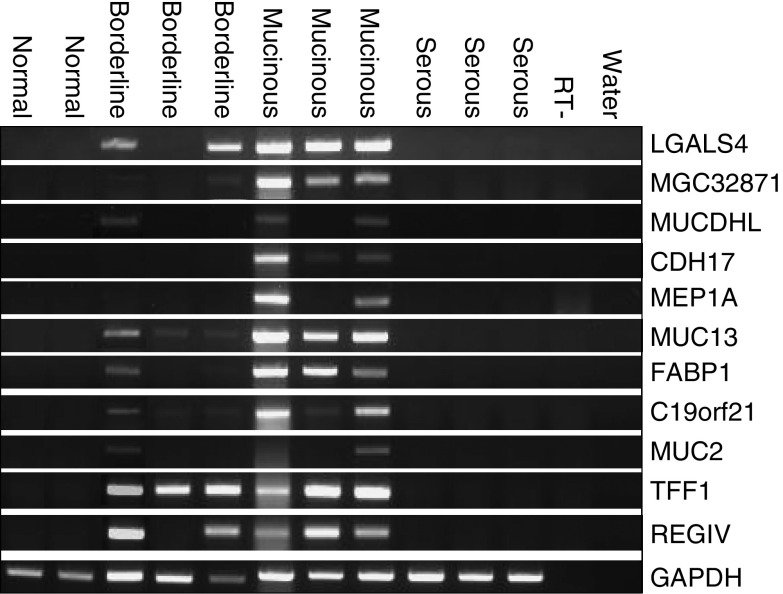
Semi-quantitative RT–PCR analysis of RNA expression in normal ovaries (*n*=2), mucinous borderline tumours (*n*=3), mucinous ovarian cancers (*n*=3) and serous ovarian cancers (*n*=3). RT-, no reverse transcriptase control; water, no cDNA. For gene descriptions, see Table 2 and Supplementary Data.

Using RT–PCR, we determined the expression patterns of 11 selected genes in RNA extracts from whole normal ovaries, mucinous borderline tumours and ovarian cancers (Figure 2). All of the genes were confirmed as being upregulated in MOC compared to serous ovarian cancer and/or normal ovaries.

### MOC express genes associated with mucin production and intestinal-type epithelium

Using Gene Ontology classifiers, we grouped the genes with upregulated expression in MOC compared to the other subtypes to identify biological processes that may specifically underlie the development and progression of MOC. Consistent with its morphological phenotype, we identified genes encoding several mucins including *MUC2*, *MUC3A* (*MUC3*) and *MUC17* but not *MUC16* (CA125). This mucin profile is similar to that of mucinous colon carcinomas, in particular the presence of *MUC2* and absence of *MUC5A* (Byrd and Bresalier, 2004; Hart, 2005). Several mucin-related molecules involved in carbohydrate metabolism and protein glycosylation were identified including *FUT3*, *GCNT3*, *SI*, *FBP1*, *UGT1A9*; and *TFF1*, an estrogen-regulated member of the trefoil factor family of secreted peptides associated with mucin production and frequently overexpressed in other mucinous adenocarcinomas (Emami *et al*, 2004).

We also identified a number of genes associated with intestinal expression including the caudal type homeobox transcription factors *CDX1* and *CDX2*. CDX transcription factors are essential in intestinal epithelial development, and are also associated with oncogenesis via the modulation of various cellular processes including proliferation, apoptosis, and cell adhesion (Guo *et al*, 2004). Moreover, two *CDX2* intestinal-specific targets were identified: sucrase isomaltase (SI), a critical gene in intestinal development (Guo *et al*, 2004) and *CDH17*, an enterocyte-specific cell adhesion molecule (Hinoi *et al*, 2002). Other intestinal-type cell adhesion molecules included *LGALS4*, a member of the galectin family of carbohydrate-binding molecules (Huflejt and Leffler, 2004); three members of the transmembrane 4 (tetraspanin) superfamily (*TM4SF4*/*IL-TMP*, *TM4SF5*/*L6H* and *TM4SF3*/*CO-029*) associated with cellular proliferation, adhesion, motility, and tumour cell metastasis (Wright *et al*, 2000); and two members of the carcinoembryonic antigen family, *CEACAM6* and *CEACAM5* (CEA), frequently expressed by at least a subset of MOC (McCluggage, 2000). The identification of intestinal-type adhesion factors suggests that altered cell adhesion is a feature of MOC, similar to other histological subtypes of ovarian cancer (Heinzelmann-Schwarz *et al*, 2004). Moreover, several of these adhesion factors have been previously implicated in carcinogenesis, including TM4SF and CEACAM family members (Scholzel *et al*, 2000; Wright *et al*, 2000; Ilantzis *et al*, 2002), CDH17 (Grotzinger *et al*, 2001; Takamura *et al*, 2004), and LGALS4 (Huflejt and Leffler, 2004).

### Cellular pathways underlying MOC development

Gene Ontology analysis identified a number of genes involved in cellular processes associated with cancer, including cell adhesion, signalling, proliferation, and apoptosis (Table 4). Several putative oncogenes were differentially expressed in MOC, including the breast tumour kinase *BRK* (*PTK6*) (Barker *et al*, 1997) not previously implicated in ovarian cancer pathogenesis; and *MST1R*/*RON*, a receptor tyrosine kinase associated with proliferation and motility of cancer cells including ovarian carcinoma (Hess *et al*, 2003; Maggiora *et al*, 2003; Wang *et al*, 2003).

Although *KRAS* mutations are associated with MOC (Shih and Kurman, 2004), we did not find any evidence of increased KRAS activity at the transcriptional level. Using GenMAPP analysis, we examined if any probesets corresponding to other members of the mitogen activated protein (MAP) kinase cascade were differentially expressed in MOC compared to the other subtypes of ovarian cancer. This revealed a slight increase in ERK1 (1.19-fold change, unadjusted *P*<0.001) and a two-fold decrease in MAP kinase kinase 1 (MEKK1) expression (0.61-fold change, unadjusted *P*=0.03), the latter being linked to cisplatin-resistance in ovarian cancer (Gebauer *et al*, 2000), a feature of MOC (Hess *et al*, 2004).

### LGALS4 is specifically expressed in MOC

**Figure 3:**
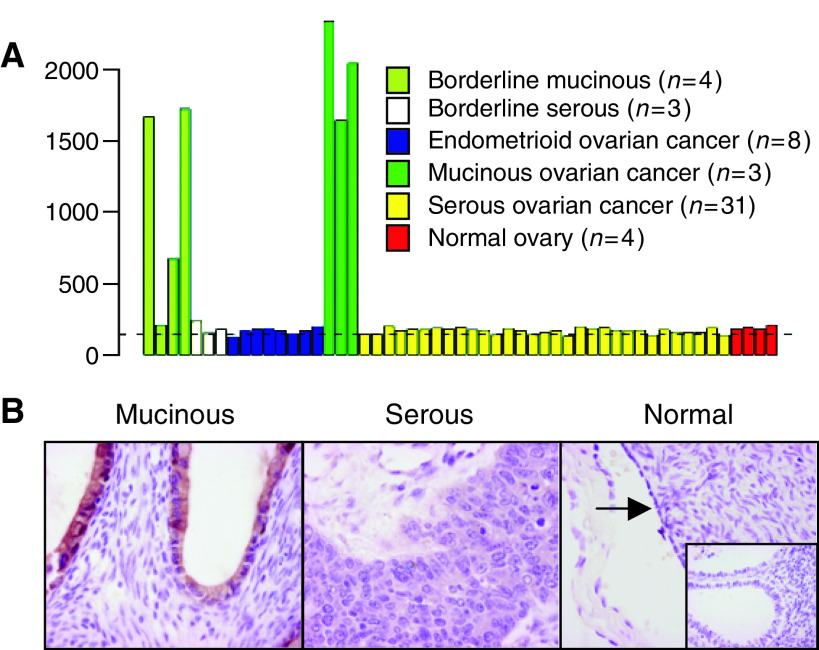
(**A**) mRNA transcript profile for LGALS4. The dashed line represents the signal intensity of the 15th percentile of the gene expression in normal body tissues (Henshall *et al*, 2003a); (**B**) representative immunohistochemistry staining for LGALS4 in MOC, serous ovarian cancer, normal ovarian surface epithelium (arrowed) and epithelial inclusion cysts (inset); × 40 magnification.

**Figure 4:**
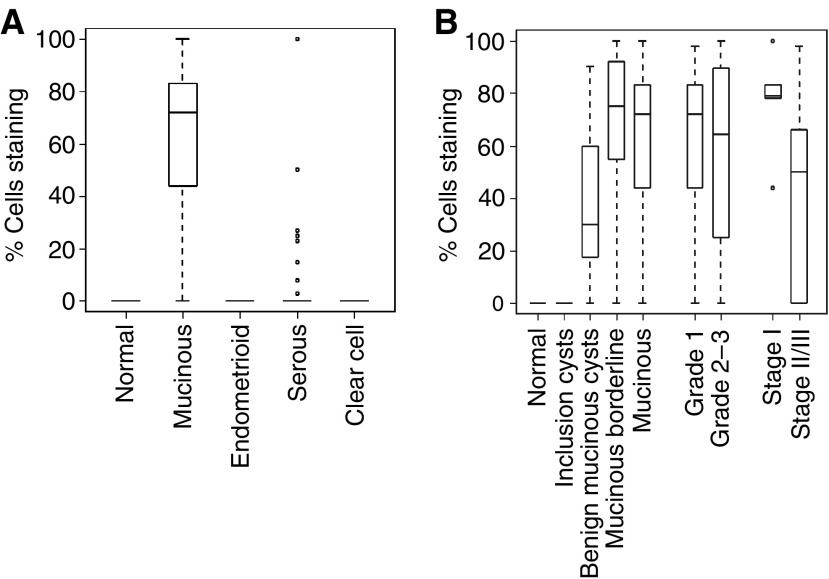
Box plots showing distribution of expression of LGALS4 in (**A**) normal ovarian surface epithelium (*n*=14) and in different histological subtypes of ovarian carcinoma: MOC (*n*=10); endometrioid ovarian cancer (*n*=22); serous ovarian cancer (*n*=55); clear cell ovarian cancer (*n*=8); and (**B**) in epithelial ovarian inclusion cysts (*n*=8); benign mucinous cysts (*n*=8); mucinous borderline tumours (*n*=29); low- (grade 1; *n*=6) and high-grade (grade 2–3; *n*=4) MOC; and low- (stage I; *n*=5) and high-stage MOC (stage II–III; *n*=5). For explanation of box plots, see Materials and Methods.

LGALS4 is an intestinal cell surface adhesion molecule that is overexpressed in intestinal carcinomas (Grotzinger *et al*, 2001). The results of the transcript profiling experiment suggested that LGALS4 was also highly overexpressed in MOC (Table 2, Figure 3A). Moreover, *LGALS4* is located at 19q13.3, a region associated with a high frequency of loss of heterozygosity in MOC (Feltmate *et al*, 2005) and where we identified a cluster of upregulated genes. We therefore examined the expression of LGALS4 in ovarian carcinoma using immunohistochemistry (Table 1, Figure 3B). In accordance with the transcript profiling results, expression of LGALS4 was highly and specifically expressed in MOC (median expression 72% of cells staining positive) compared to the other ovarian carcinoma subtypes (serous and endometroid, *P*<0.001; clear cell *P*=0.002) and to normal ovarian surface epithelium (*P*=0.002), all of which had a median expression equivalent to zero (Figure 4A). To identify if LGALS4 expression occurs early in disease onset, we examined its expression in benign mucinous cysts and mucinous borderline tumours, in addition to low- and high-stage MOC. LGALS4 expression was detected at a median expression level of approximately 30% of cells staining in benign mucinous cysts, increasing in borderline tumours to similar levels of expression as in MOC (>70% of cells; Figure 4B). There was no significant difference in expression between borderline tumours and low grade MOC (*P*=0.47), and although a decrease in expression from low- to high-stage MOC was observed, this was not statistically significant (*P*=0.21). Statistical analysis did not reveal any correlation between LGALS4 expression and clinicopathological parameters (age, grade, stage, outcome; Table 1) in the ovarian mucinous tumour cohort (data not shown).

## DISCUSSION

Mucinous ovarian cancers are one of the less common histological subtypes of ovarian carcinoma. Combined with the difficulty in accurate diagnosis of primary disease, its relative rarity has contributed to the lack of knowledge regarding the molecular basis of its development and progression. In this study, we have shown that MOC show a gene expression profile that both overlaps with and is distinct from the other histological subtypes of ovarian carcinoma, presumably reflecting their common ovarian origin but different morphological and clinical presentations. In particular, we found that MOC express genes that underlie their morphological phenotype, including intestinal-specific genes, which likely reflects the intestinal-type differentiation characteristic of most MOC (Feeley and Wells, 2001). A variety of mucin molecules are also expressed in MOC. Alterations in expression of mucins, including loss of organ specificity, are a common feature of cancer and are associated with altered biological properties including metastatic potential (Byrd and Bresalier, 2004). In addition, variations in mucin glycosylation patterns can cause changes in tumour cell adhesion, migration and invasion (Casey *et al*, 2003), and can be mediated by the differential expression of glycosylation enzymes including fucosyltransferases and sialyltransferases. We identified several such enzymes, suggesting specific mucin glycosylation patterns are a feature of MOC.

It is likely that many carcinomas diagnosed as primary mucinous ovarian cancer are more likely to be metastatic disease originating in the gastrointestinal tract (Gilks, 2004; Seidman *et al*, 2004). In this study, we were particularly careful to only include patients that clearly fit with current clinical and histological guidelines as primary MOC rather than metastatic disease (Seidman *et al*, 2003; Hart, 2005). However, these strict selection criteria, combined with the relative rarity of MOC, resulted in a small sample number both for the transcript profiling and validation experiments. In addition, although comprising at least 75% tumour cells, the tissue samples used in the transcript profiling experiments were not microdissected and therefore may contain a small proportion of stromal elements. Therefore, our results remain to be validated in independent studies. To this end, several published studies have reported similar findings in regard to the genetic profile of MOC. First, Schwartz *et al* (2002) used principal components analysis to show that gene expression profiles could distinguish MOC from serous ovarian cancer, with some overlap with endometrioid ovarian cancer. Secondly, using cDNA arrays incorporating 9121 elements, Ono *et al* (2000) identified 115 genes that were differentially regulated between serous ovarian carcinomas and MOC). By comparing the Unigene/Locus Link identifiers corresponding to the GenBank accessions in the Ono study to the gene identifiers in our study (Table 2 and Supplementary Data), we identified only one gene (*TUBB2*; tubulin beta 2) that overlaps between our lists of differentially expressed genes. A more recent study reported 46 genes that were overexpressed in MOC compared to the other histological subtypes of ovarian carcinoma and to normal ovarian surface epithelium (Marquez *et al*, 2005). Fifteen of those genes (*TM4SF3*, *S100P*, *TM4SF4*, *CEACAM6*, *LGALS4*, *CEACAM5*, *TUBB*, *CTSE*, *GCNT3*, *REG4*, *FABP1*, *SDCBP2*, *TFF1*, *RNF128*, *PLAC8*) were also identified in our study. Moreover, we also showed that LGALS4 is consistently highly expressed in MOC but is absent in the other histological subtypes of ovarian cancer and normal ovaries using immunohistochemistry, thus confirming the transcript profiling results.

Progression from borderline tumours and low-stage carcinoma to advanced MOC is associated with a poor outcome; hence the identification of tumour markers that can detect early disease, together with those that can predict patients likely to progress to advanced stage MOC, would have a major impact on patient prognosis. We determined that LGALS4 is not expressed in normal ovarian surface epithelium but is expressed at high levels in mucinous borderline tumours and in benign mucinous cysts, consistent with activation of expression early in MOC development. We did not identify any genes including *LGALS4* that were significantly differentially expressed between mucinous borderline tumours and MOC, suggesting that there may be very few or only subtle changes in gene expression between mucinous borderline tumours and low-stage MOC (which were used in the transcript profiling analysis), concordant with their similar outcomes. A study with sufficient power to compare high-stage MOC to borderline and low-stage MOC may reveal gene expression changes that correlate with the poor prognosis in these patients.

There are currently no specific or sensitive serum markers for the diagnosis of MOC (Rapkiewicz *et al*, 2004). MOC often fail to express the ovarian cancer serum marker CA125 (MUC16), which is frequently elevated in the serum of patients with nonmucinous ovarian carcinoma. Although a cell surface adhesion molecule, LGALS4 has at least a partial extracellular component (Huflejt and Leffler, 2004), but to our knowledge there is no report of its presence in serum. Serum antibodies against LGALS4 have, however, been reported in a patient with colorectal cancer (Scanlan *et al*, 1998). Given the high level of LGALS4 expression in MOC, one might predict that similar antibodies could be detected in patients with MOC, which is currently under investigation. Hence LGALS4 may have application as an early serum diagnostic marker of MOC, either alone or in combination with other markers such as CEA (CEACAM5) and CA19.9 (Rapkiewicz *et al*, 2004; Hart, 2005). In addition, the high level of LGALS4 expression in MOC may aid in the histological differentiation of primary MOC from metastatic ovarian carcinoma arising at other sites (Heinzelmann-Schwarz *et al*; manuscript submitted for publication).

As previously suggested (Hess *et al*, 2004), the obvious genetic similarities of MOC with mucinous-type intestinal carcinomas support a move toward the use of a therapeutic approach tailored to the molecular characteristics of MOC rather than the tissue of origin. Patients with advanced stage MOC generally receive the same adjuvant chemotherapy as the other subtypes of ovarian carcinoma, normally a platinum-based approach combined with paclitaxel. The poor survival associated with advanced MOC may reflect a failure to respond to this regime (Hess *et al*, 2004). Hence, alternative combination chemotherapy regimes that target both the ovarian and mucinous intestinal genetic components of MOC, such as a platin compound combined with 5-fluorouracil, commonly used in the treatment of intestinal carcinomas, may prove to be more efficacious for MOC. This, however, remains to be tested in appropriate clinical trials.

## Figures and Tables

**Table 1:** Clinical and pathological details of the ovarian tumour cohort used for immunohistochemistry (*n*=124)^a^

**Variable**	**No. patients (%)**
*Histological type*	
Serous	55 (44.4)
Mucinous	39 (31.4)
Endometrioid	22 (17.7)
Clear cell	8 (6.5)
	
*FIGO stage* b	
I	27 (28.4)
II	5 (5.3)
III	55 (57.9)
IV	8 (8.4)
	
*Grade* c	
Borderline (mucinous only)	29 (24.8)
1	20 (17.1)
2	35 (29.9)
3	33 (28.2)
	
*Age*	
<60	62 (50.0)
⩾60	62 (50.0)
	
*Residual disease*	
⩽1	92 (74.2)
>1	32 (25.8)
	
*CA125* d	
⩽500	58 (60.0)
>500	39 (40.0)
	
*Outcome* c	
Alive	62 (53.0)
Death (related to malignancy)	48 (41.0)
Death (unrelated or unknown cause)	7 (6.0)

a Unless otherwise stated.

b Carcinomas only; *n*=95.

c *n*=117.

d *n*=97.

**Table 2:** Genes (*n*=50 of 167 probesets^a^) identified as upregulated in MOC compared to other histological subtypes of ovarian cancer (ranked by adjusted *P*<0.01)

**Rank**	**Symbol^b^**	**Name**	**Unigene^c^**	**Locus link**	**Location**
1	LGALS4	Lectin, galactoside-binding, soluble, 4 (galectin 4)	Hs.5302	3960	19q13.2
2		cDNA clone IMAGE:5759948, partial cds	Hs.447537	NA	15q15.1
3		Hypothetical protein MGC32871	Hs.242014	119467	10q26.3
4	MUCDHL	Mucin and cadherin-like	Hs.165619	53841	11p15.5
5		apobec-1 complementation factor	Hs.8349	29974	10q21.1
6	CDH17	Cadherin 17, LI cadherin (liver-intestine)	Hs.89436	1015	8q22.1
7	MEP1A	Meprin A, alpha (PABA peptide hydrolase)	Hs.179704	4224	6p12-p11
8	MUC13	Mucin 13, epithelial transmembrane	Hs.5940	56667	3q21
9	FABP1	Fatty acid binding protein 1, liver	Hs.380135	2168	2p11
10	MUC3B	Mucin 3B, intestinal	Hs.489354	NA	7q22
11	CEACAM5	CEA-related cell adhesion molecule 5 (CEA)	Hs.220529	1048	19q13.2
12	PDZK2	PDZ domain containing 2	Hs.374726	79849	11q23.3
13	GPA33	Glycoprotein A33 (transmembrane)	Hs.437229	10223	1q24.1
14	RNF128	Ring finger protein 128	Hs.496542	79589	Xq22.3
15	MUCDHL	Mucin and cadherin-like	Hs.165619	53841	11p15.5
16	EPS8L3	EPS8-like 3	Hs.485352	79574	1p13.2
17	BCL2L14	BCL2-like 14 (apoptosis facilitator)	Hs.504794	79370	12p13.2
18	SYTL2	Synaptotagmin-like 2, transcript variant a	Hs.369520	54843	11q14.1
19	HMGCS2	3-hydroxy-3-methylglutaryl-Coenzyme A synthase 2 (mitochondrial)	Hs.59889	3158	1p13-p12
20	FAM3D	Family with sequence similarity 3, member D	Hs.61265	131177	3p21.2
21	MYO1A	Myosin IA	Hs.5394	4640	12q13.3
22	SLC26A3	Solute carrier family 26, member 3	Hs.1650	1811	7q31
23	ATP10B	ATPase, Class V, type 10B	Hs.109358	23120	5q34
24	BTNL8	Butyrophilin-like 8	Hs.189109	79908	5q35.3
25	MYO7B	Myosin VIIB	Hs.154578	4648	2q14.3
26	CDX1	Caudal type homeo box transcription factor 1	Hs.1545	1044	5q33.1
27		cDNA clone IMAGE:4661388, partial cds	Hs.306721	400573	17p13.1
28		ESTs; moderate similarity to protein P39188 (H.sapiens)	Hs.282795	NA	10q11.23
29		ESTs	Hs.116462	NA	20q13.12
30		Cisplatin resistance associated	Hs.425144	10903	1q21.2
31	TRIM31	Tripartite motif-containing 31	Hs.493275	11074	6p21.3
32	UGT1A9	UDP glycosyltransferase 1 family, polypeptide A9	Hs.124112	54600	2q37
33	ACHE	Acetylcholinesterase (YT blood group)	NA	43	7q22
34	PLA2G10	Phospholipase A2, group X	Hs.144442	401831	16p13.1-p12
35		Hypothetical protein LOC144347	Hs.432901	144347	12q24.31
36	GUCY2C	Guanylate cyclase 2C (heat stable enterotoxin receptor)	Hs.524278	2984	12p12
37		cDNA clone IMAGE:4806358, partial cds	Hs.328236	NA	4q32.3
38	PLAC8	Placenta-specific 8	Hs.371003	51316	4q21.3
39		KIAA0828 protein	Hs.195058	23382	7q32.3
40	COL17A1	Collagen, type XVII, alpha 1	Hs.117938	1308	10q25.1
41	CLCA1	Chloride channel, calcium activated, family member 1	Hs.194659	1179	1p31-p22
42		Hypothetical protein FLJ20225	Hs.124835	54546	1p36.13
43	TM4SF4	Transmembrane 4 superfamily member 4	Hs.133527	7104	3q25
44	MUC17	mucin 17	Hs.271819	140453	7q22
45	FA2H	Fatty acid 2-hydroxylase	Hs.461329	79152	16q23
46	HNF4A	Hepatocyte nuclear factor 4, alpha	Hs.116462	3172	20q12-q13.1
47	SEMA4G	Sema domain, immunoglobulin domain, transmembrane domain and short cytoplasmic domain, (semaphorin) 4G	Hs.444359	57715	10q24.32
48		cDNA FLJ26898 fis, clone RCT00475	Hs.199371	NA	11q13.1
49	C9orf152	Chromosome 9 open reading frame 152	Hs.125608	401546	9q32
50	S100P	S100 calcium binding protein P	Hs.2962	6286	4p16

a Full list of genes are listed in Supplementary Data.

b HUGO.

c Unigene identifiers were derived from the UniGene Build #176 (October 2004).

**Table 3:** Genes identified as (A) up-regulated (*n*=40) and (B) down-regulated (*n*=4) in MOC compared to normal ovaries (ranked by adjusted *P*<0.01). Genes highlighted in bold (*n*=13) are also up-regulated in MOC compared to other subtypes of ovarian cancer

**Rank**	**Symbol**	**Name**	**Unigene**	**Locus Link**	**Location**
(A) *Upregulated*					
1	**LGALS4**	**Lectin, galactoside-binding, soluble, 4 (galectin 4**)	Hs.5302	3960	19q13.2
2		Hypothetical protein FLJ20171	Hs.487471	54845	8q22.1
3	TACSTD1	Tumor-associated calcium signal transducer 1 (Ep-CAM)	Hs.692	4072	2p21
4	ERBB3	v-erb-b2 erythroblastic leukemia viral oncogene homolog 3	Hs.306251	2065	12q13
5		**cDNA clone IMAGE:5759948, partial cds**	Hs.447537	NA	15q15.1
6	BCMP11	Breast cancer membrane protein 11	Hs.100686	155465	7p21.1
7	CXXC5	CXXC finger 5	Hs.189119	51523	5q31.2
8	EHF	Ets homologous factor	Hs.502306	26298	11p12
9	STARD10	START domain containing 10	Hs.188606	10809	11q13
10	**C19orf21**	**Chromosome 19 open reading frame 21**	Hs.439180	126353	19p13.3
11	**MUC13**	**Mucin 13, epithelial transmembrane**	Hs.5940	56667	3q21.2
12	PROM1	Prominin 1	Hs.479220	8842	4p15.32
13	**MUCDHL**	**Mucin and cadherin-like**	Hs.165619	53841	11p15.5
14		Hypothetical gene supported by BC022385; BC035868; BC048326	Hs.390599	440335	16p13.3
15	**CEACAM5**	**Carcinoembryonic antigen-related cell adhesion molecule 5 (CEA)**	Hs.220529	1048	19q13.1-q13.2
16	AGPAT2	1-acylglycerol-3-phosphate O-acyltransferase 2	Hs.320151	10555	9q34.3
17		Hypothetical protein MGC32871	Hs.242014	119467	10q26.2
18		Hypothetical protein MGC11242	Hs.368260	79170	17q21.32
19	CLDN7	Claudin 7	Hs.513915	1366	17p13
20		FLJ46072 protein	Hs.67776	286077	8q24.3
21	**CALML4**	**Calmodulin-like 4; breast cancer antigen NY-BR-20**	Hs.435457	91860	15q23
22	PLS1	Plastin 1 (I isoform)	Hs.203637	5357	3q23
23	KLF5	Kruppel-like factor 5 (intestinal)	Hs.508234	688	13q22.1
24	KIAA0101	KIAA0101	Hs.81892	9768	15q22.31
25	TSPAN-1	Tetraspan 1	Hs.38972	10103	1p34.1
26	TDE2L	Tumor differentially expressed 2-like	Hs.270655	347735	1p35.1
27	AGR2	Anterior gradient 2 homolog (Xenopus laevis)	Hs.530009	10551	7p21.3
28	**ARHGAP27**	**Rho GTPase activating protein 27**	Hs.463165	201176	17q21.31
29	**NMES1**	**Normal mucosa of esophagus specific 1**	Hs.112242	84419	15q21.1
30	CEACAM1	Carcinoembryonic antigen-related cell adhesion molecule 1	Hs.512682	634	19q13.2
31	ST14	Suppression of tumorigenicity 14 (colon carcinoma, matriptase, epithin)	Hs.504315	6768	11q24-q25
32		LOC387882 hypothetical protein	Hs.525657	387882	12q23.3
33	TRPM4	Transient receptor potential cation channel, subfamily M, member 4	Hs.467101	54795	19q13.33
34	**BCLP**	**Beta-casein-like protein**	Hs.534521	113452	1p35-p34
35	IFI30	Interferon, gamma-inducible protein 30	Hs.14623	10437	19p13.1
36	ABP1	Amiloride binding protein 1 (amine oxidase (copper-containing))	Hs.521296	26	7q34-q36
37	ERBB3	v-erb-b2 erythroblastic leukemia viral oncogene homolog 3	Hs.306251	2065	12q13
38	MAL2	Mal, T-cell differentiation protein 2	Hs.201083	114569	8q24.12
39	**CDH17**	**Cadherin 17, LI cadherin (liver-intestine)**	Hs.89436	1015	8q22.1
40	CDH1	Cadherin 1, type 1, E-cadherin (epithelial)	Hs.461086	999	16q22.1
					
(B) *Downregulated*					
1		Similar to lymphocyte antigen 6 complex, locus G5B; G5b protein	Hs.23650	112476	16p11.2
2	RARRES2	Retinoic acid receptor responder (tazarotene induced) 2 (TIG2)	Hs.521286	5919	7q36.1
3	PDGFD	Platelet derived growth factor D	Hs.352298	80310	11q22.3
4		Hypothetical protein MGC1136	Hs.8719	78986	8p12

**Table 4:** Selected categories/genes from Gene Ontology (GO) analysis of genes specifically upregulated in MOC compared to other histological subtypes of ovarian cancer

**Rank**	**Symbol**	**Name**	**Locus link**
*Biological process*			
Cell adhesion			
1	LGALS4	Lectin, galactoside-binding, soluble, 4 (galectin 4)	3960
6	CDH17	Cadherin 17, LI cadherin (liver-intestine)	1015
11	CEACAM5	CEA-related cell adhesion molecule 5 (CEA)	1048
61	CEACAM6	CEA-related cell adhesion molecule 6	4680
			
Cell signalling			
103	MST1R	Macrophage stimulating 1 receptor (c-met-related tyrosine kinase) (RON)	4486
113	PTK6	PTK6 protein tyrosine kinase 6; breast tumor kinase BRK	5753
			
Proliferation			
43	TM4SF4	Transmembrane 4 superfamily member 4	7104
72	TM4SF5	Transmembrane 4 superfamily member 5	9032
103	MST1R	Macrophage stimulating 1 receptor (c-met-related tyrosine kinase) (RON)	4486
122	TM4SF3	Transmembrane 4 superfamily member 3	7103
			
Apoptosis			
17	BCL2L14	BCL2-like 14 (apoptosis facilitator)	79370
			
*Molecular function*			
Mucins			
4	MUCDHL	Mucin and cadherin-like	53841
8	MUC13	Mucin 13, epithelial transmembrane	56667
10	MUC3B	Mucin 3A, intestinal	NA
15	MUCDHL	Mucin and cadherin-like	53841
44	MUC17	Mucin 17	140453
51	MUC2	Mucin 2, intestinal/tracheal	4583
			
Carbohydrate metabolism			
54	TFF1	Trefoil factor 1 (breast cancer, estrogen-inducible sequence)	7031
59	FUT3	Fucosyltransferase 3 (galactoside 3(4)-L-Fucosyltransferase, Lewis blood group)	2525
74	SI	Sucrase-isomaltase (alpha-glucosidase)	6476
85	GCNT3	Glucosaminyl (*N*-acetyl) transferase 3, mucin type	9245
			
Protein glycosylation			
32	UGT1A9	UDP glycosyltransferase 1 family, polypeptide A9	54600
59	FUT3	Fucosyltransferase 3 (galactoside 3(4)-L-Fucosyltransferase, Lewis blood group)	2525
79	FBP1	Fructose-1,6-bisphosphatase 1	2203
85	GCNT3	Glucosaminyl (*N*-acetyl) transferase 3, mucin type	9245
			
Protein hydrolysis			
7	MEP1A	Meprin A, alpha (PABA peptide hydrolase)	4224
			
Transcription factors			
26	CDX1	Caudal type homeo box transcription factor 1	1044
46	HNF4A	Hepatocyte nuclear factor 4, alpha	3172
135	CDX2	Caudal type homeo box transcription factor 2	1045
