# Supplementary material for: A distinct molecular profile associated with mucinous epithelial ovarian cancer
Source: Br J Cancer. 2006 Feb 28;94(6):904–13. doi: 10.1038/sj.bjc.6603003 (PMC2361366; doi:10.1038/sj.bjc.6603003)
Supplement: Supplementary Table 2 [file 94-6603003x1.doc]

**Supplementary Data: Table 2 (full)**

**A. Up-regulated genes in MOC compared to other subtypes of ovarian carcinoma**

| **Rank** | **Symbol** | Name | **Unigene** | **Locus Link** | **Location** |
| --- | --- | --- | --- | --- | --- |
| 1 | LGALS4 | lectin, galactoside-binding, soluble, 4 (galectin 4) | Hs.5302 | 3960 | 19q13.2 |
| 2 |  | cDNA clone IMAGE:5759948, partial cds | Hs.447537 | NA | 15q15.1 |
| 3 |  | hypothetical protein MGC32871 | Hs.242014 | 119467 | 10q26.3 |
| 4 | MUCDHL | mucin and cadherin-like | Hs.165619 | 53841 | 11p15.5 |
| 5 | ACF | apobec-1 complementation factor | Hs.8349 | 29974 | 10q21.1 |
| 6 | CDH17 | cadherin 17, LI cadherin (liver-intestine) | Hs.89436 | 1015 | 8q22.1 |
| 7 | MEP1A | meprin A, alpha (PABA peptide hydrolase) | Hs.179704 | 4224 | 6p12-p11 |
| 8 | MUC13 | mucin 13, epithelial transmembrane | Hs.5940 | 56667 | 3q21 |
| 9 | FABP1 | fatty acid binding protein 1, liver | Hs.380135 | 2168 | 2p11 |
| 10 | MUC3B | mucin 3B, intestinal | Hs.489354 | NA | 7q22 |
| 11 | CEACAM5 | CEA-related cell adhesion molecule 5 (CEA) | Hs.220529 | 1048 | 19q13.2 |
| 12 | PDZK2 | PDZ domain containing 2 | Hs.374726 | 79849 | 11q23.3 |
| 13 | GPA33 | glycoprotein A33 (transmembrane) | Hs.437229 | 10223 | 1q24.1 |
| 14 | RNF128 | ring finger protein 128 | Hs.496542 | 79589 | Xq22.3 |
| 15 | MUCDHL | mucin and cadherin-like | Hs.165619 | 53841 | 11p15.5 |
| 16 | EPS8L3 | EPS8-like 3 | Hs.485352 | 79574 | 1p13.2 |
| 17 | BCL2L14 | BCL2-like 14 (apoptosis facilitator) | Hs.504794 | 79370 | 12p13.2 |
| 18 | SYTL2 | synaptotagmin-like 2, transcript variant a | Hs.369520 | 54843 | 11q14.1 |
| 19 | HMGCS2 | 3-hydroxy-3-methylglutaryl-Coenzyme A synthase 2 (mitochondrial) | Hs.59889 | 3158 | 1p13-p12 |
| 20 | FAM3D | family with sequence similarity 3, member D | Hs.61265 | 131177 | 3p21.2 |
| 21 | MYO1A | myosin IA | Hs.5394 | 4640 | 12q13.3 |
| 22 | SLC26A3 | solute carrier family 26, member 3 | Hs.1650 | 1811 | 7q31 |
| 23 | ATP10B | ATPase, Class V, type 10B | Hs.109358 | 23120 | 5q34 |
| 24 | BTNL8 | butyrophilin-like 8 | Hs.189109 | 79908 | 5q35.3 |
| 25 | MYO7B | myosin VIIB | Hs.154578 | 4648 | 2q14.3 |
| 26 | CDX1 | caudal type homeo box transcription factor 1 | Hs.1545 | 1044 | 5q33.1 |
| 27 |  | cDNA clone IMAGE:4661388, partial cds | Hs.306721 | 400573 | 17p13.1 |
| 28 |  | ESTs; moderate similarity to protein sp:P39188 (H.sapiens) | Hs.282795 | NA | 10q11.23 |
| 29 |  | ESTs | Hs.116462 | NA | 20q13.12 |
| 30 | CRA | cisplatin resistance associated | Hs.425144 | 10903 | 1q21.2 |
| 31 | TRIM31 | tripartite motif-containing 31 | Hs.493275 | 11074 | 6p21.3 |
| 32 | UGT1A9 | UDP glycosyltransferase 1 family, polypeptide A9 | Hs.124112 | 54600 | 2q37 |
| 33 | ACHE | acetylcholinesterase (YT blood group) | NA | 43 | 7q22 |
| 34 | PLA2G10 | phospholipase A2, group X | Hs.144442 | 401831 | 16p13.1-p12 |
| 35 |  | hypothetical protein LOC144347 | Hs.432901 | 144347 | 12q24.31 |
| 36 | GUCY2C | guanylate cyclase 2C (heat stable enterotoxin receptor) | Hs.524278 | 2984 | 12p12 |
| 37 |  | cDNA clone IMAGE:4806358, partial cds | Hs.328236 | NA | 4q32.3 |
| 38 | PLAC8 | placenta-specific 8 | Hs.371003 | 51316 | 4q21.3 |
| 39 |  | KIAA0828 protein | Hs.195058 | 23382 | 7q32.3 |
| 40 | COL17A1 | collagen, type XVII, alpha 1 | Hs.117938 | 1308 | 10q25.1 |
| 41 | CLCA1 | chloride channel, calcium activated, family member 1 | Hs.194659 | 1179 | 1p31-p22 |
| 42 |  | hypothetical protein FLJ20225 | Hs.124835 | 54546 | 1p36.13 |
| 43 | TM4SF4 | transmembrane 4 superfamily member 4 | Hs.133527 | 7104 | 3q25 |
| 44 | MUC17 | mucin 17 | Hs.271819 | 140453 | 7q22 |
| 45 | FA2H | fatty acid 2-hydroxylase | Hs.461329 | 79152 | 16q23 |
| 46 | HNF4A | hepatocyte nuclear factor 4, alpha | Hs.116462 | 3172 | 20q12-q13.1 |
| 47 | SEMA4G | sema domain, immunoglobulin domain (Ig), transmembrane domain (TM) and short cytoplasmic domain, (semaphorin) 4G | Hs.444359 | 57715 | 10q24.32 |
| 48 |  | cDNA FLJ26898 fis, clone RCT00475 | Hs.199371 | NA | 11q13.1 |
| 49 | C9orf152 | chromosome 9 open reading frame 152 | Hs.125608 | 401546 | 9q32 |
| 50 | S100P | S100 calcium binding protein P | Hs.2962 | 6286 | 4p16 |
| 51 | MUC2 | mucin 2, intestinal/tracheal | Hs.315 | 4583 | 11p15.5 |
| 52 |  | hypothetical protein FLJ23654 | Hs.443728 | NA | 5q32 |
| 53 | DDC | dopa decarboxylase (aromatic L-amino acid decarboxylase) | Hs.359698 | 1644 | 7p11 |
| 54 | TFF1 | trefoil factor 1 (breast cancer, estrogen-inducible sequence) | Hs.162807 | 7031 | 21q22.3 |
| 55 | USH1C | usher syndrome 1C (autosomal recessive, severe) | Hs.502072 | 80102 | 11p15.1-p14 |
| 56 | GPX2 | glutathione peroxidase 2 (gastrointestinal) | Hs.2704 | 2877 | 14q24.1 |
| 57 | VILL | villin-like | Hs.103665 | 50853 | 3p21.3 |
| 58 | CTXL | cortical thymocyte receptor (X. laevis CTX) like | Hs.112377 | 23584 | 11q24 |
| 59 | FUT3 | fucosyltransferase 3 (galactoside 3(4)-L-fucosyltransferase, Lewis blood group) | Hs.169238 | 2525 | 19p13.3 |
| 60 | LOC257407 | hypothetical protein LOC257407 | Hs.526596 | 401034 | 2q37.1 |
| 61 | CEACAM6 | carcinoembryonic antigen-related cell adhesion molecule 6 | Hs.466814 | 4680 | 19q13.2 |
| 62 | SLC22A18 | solute carrier family 22 (organic cation transporter), member 18 | Hs.50868 | 5002 | 11p15.5 |
| 63 | CEACAM6 | carcinoembryonic antigen-related cell adhesion molecule 6 | Hs.466814 | 4680 | 19q13.2 |
| 64 | ALDOB | aldolase B, fructose-bisphosphate | Hs.530274 | 229 | 9q21.3-q22.2 |
| 65 | HSD17B2 | hydroxysteroid (17-beta) dehydrogenase 2 | Hs.162795 | 3294 | 16q24.1-q24.2 |
| 66 | VIL1 | villin 1 | Hs.534364 | 7429 | 2q35-q36 |
| 67 | BCLP | beta-casein-like protein | Hs.534521 | 113452 | 1p35-p34 |
| 68 | CES2 | carboxylesterase 2 (intestine, liver) | Hs.282975 | 8824 | 16q22.1 |
| 69 | CFTR | cystic fibrosis transmembrane conductance regulator, ATP-binding cassette (sub-family C, member 7) | Hs.489786 | 1080 | 7q31.2 |
| 70 | VIPR1 | vasoactive intestinal peptide receptor 1 | Hs.348500 | 7433 | 3p22 |
| 71 | CENTA1 | centaurin, alpha 1 | Hs.135183 | 11033 | 7p22.3 |
| 72 | TM4SF5 | transmembrane 4 superfamily member 5 | Hs.184194 | 9032 | 17p13.3 |
| 73 | RNASE4 | ribonuclease, RNase A family, 5 | Hs.283749 | 283 | 14q11.1 |
| 74 | SI | sucrase-isomaltase (alpha-glucosidase) | Hs.429596 | 6476 | 3q25.2-q26.2 |
| 75 | CLDN15 | claudin 15 | Hs.38738 | 24146 | 7q22.1 |
| 76 | SLC27A2 | solute carrier family 27 (fatty acid transporter), member 2 | Hs.11729 | 11001 | 15q21.2 |
| 77 |  | NA | NA | NA | 1p21 |
| 78 | FLJ20200 | hypothetical protein FLJ20200 | Hs.165803 | 54854 | 19q13.33 |
| 79 | FBP1 | fructose-1,6-bisphosphatase 1 | Hs.494496 | 2203 | 9q22.3 |
| 80 | ATP2A3 | ATPase, Ca++ transporting, ubiquitous | Hs.513870 | 489 | 17p13.3 |
| 81 | PLEKHJ1 | pleckstrin homology domain containing, family J member 1 | Hs.115232 | 55111 | 19p13.3 |
| 82 | PPP1R14D | protein phosphatase 1, regulatory (inhibitor) subunit 14D | Hs.192927 | 54866 | 15q14 |
| 83 |  | ESTs; moderate similarity to protein sp:P39195 | Hs.152307 | NA | 15q22.2 |
| 84 | ITPKA | inositol 1,4,5-trisphosphate 3-kinase A | Hs.2722 | 3706 | 15q14-q21 |
| 85 | GCNT3 | glucosaminyl (N-acetyl) transferase 3, mucin type | Hs.194710 | 9245 | 15q21.3 |
| 86 | SDCBP2 | syndecan binding protein (syntenin) 2 | Hs.516836 | 27111 | 20p13 |
| 87 | SLC9A1 | solute carrier family 9 (sodium/hydrogen exchanger), isoform 1 | Hs.469116 | 6548 | 1p36.1-p35 |
| 88 | INSL3 | insulin-like 3 (Leydig cell) | Hs.37062 | 3640 | 19p13.2-p12 |
| 89 | STARD10 | START domain containing 10 | Hs.188606 | 10809 | 11q13 |
| 90 | C9orf158 | chromosome 9 open reading frame 158 | Hs.7739 | 81932 | 9q33.1 |
| 91 | PRSS1 | trypsin 1 precursor (cationic trypsinogen) | Hs.511525 | 5644 | 7q34 |
| 92 | C20orf42 | chromosome 20 open reading frame 42 | Hs.472054 | 55612 | 20p12.3 |
| 93 |  | NA | NA | NA | 11p11.2 |
| 94 | NMES1 | normal mucosa of esophagus specific 1 | Hs.112242 | 84419 | 15q15.1 |
| 95 | REG4 | regenerating islet-derived family, member 4 | Hs.171480 | 83998 | 1p13.1-p12 |
| 96 | GMDS | GDP-mannose 4,6-dehydratase | Hs.144496 | 2762 | 6p25 |
| 97 | TNRC9 | trinucleotide repeat containing 9 | Hs.460789 | 27324 | 16q12.2 |
| 98 | MAWBP | MAWD binding protein | Hs.198158 | 64081 | 10q21.3 |
| 99 | FLJ38690 | hypothetical protein FLJ38690 | Hs.528359 | 283460 | 12q24.31 |
| 100 | CYP2S1 | cytochrome P450, family 2, subfamily S, polypeptide 1 | Hs.98370 | 29785 | 19q13.1 |
| 101 |  | cDNA: FLJ21572 fis, clone COL06651 | Hs.535636 | NA | 2q37.1 |
| 102 | PPARG | peroxisome proliferative activated receptor, gamma | Hs.387667 | 5468 | 3p25 |
| 103 | MST1R | macrophage stimulating 1 receptor (c-met-related tyrosine kinase) (RON) | Hs.2942 | 4486 | 3p21.3 |
| 104 | KIAA1201 | KIAA1201 protein | Hs.347534 | 57476 | 11q24.1 |
| 105 | PRSS1 | trypsin 1 precursor (cationic trypsinogen) | Hs.511525 | 5644 | 7q34 |
| 106 | PPARG | peroxisome proliferative activated receptor, gamma | Hs.162646 | 5468 | 3p25 |
| 107 |  | clone IMAGE:3897156 | Hs.152258 | NA | 17q21.32 |
| 108 | BCAS1 | breast carcinoma amplified sequence 1 | Hs.400556 | 8537 | 20q13.2-q13.3 |
| 109 | CA2 | carbonic anhydrase II | Hs.155097 | 760 | 8q22 |
| 110 | LOC55971 | insulin receptor tyrosine kinase substrate | Hs.489237 | 55971 | 7q22.1 |
| 111 | NA | ESTs | Hs.446324 | NA | 9p13.3 |
| 112 | CYP2S1 | cytochrome P450, family 2, subfamily S, polypeptide 1 | Hs.98370 | 29785 | 19q13.1 |
| 113 | PTK6 | PTK6 protein tyrosine kinase 6/BRK | Hs.51133 | 5753 | 20q13.3 |
| 114 | LOC63928 | hepatocellular carcinoma antigen gene 520 | Hs.178589 | 63928 | 16p12.3 |
| 115 | EPB41L4B | erythrocyte membrane protein band 4.1 like 4B | Hs.269180 | 54566 | 9q31-q32 |
| 116 | C19orf21 | chromosome 19 open reading frame 21 | Hs.439180 | 126353 | 19p13.3 |
| 117 | MOGAT2 | monoacylglycerol O-acyltransferase 2 | Hs.288568 | 80168 | 11q13.3 |
| 118 | CALML4 | calmodulin-like 4 | Hs.435457 | 91860 | 15q22.31 |
| 119 | C20orf42 | chromosome 20 open reading frame 42 | Hs.472054 | 55612 | 20p12.3 |
| 120 | PRAP1 | proline-rich acidic protein 1 | Hs.15951 | 118471 | 10q26.3 |
| 121 | ABCC3 | ATP-binding cassette, sub-family C (CFTR/MRP), member 3 | Hs.463421 | 8714 | 17q22 |
| 122 | TM4SF3 | transmembrane 4 superfamily member 3 | Hs.170563 | 7103 | 12q14.1-q21.1 |
| 123 | ARHGAP27 | Rho GTPase activating protein 27 | Hs.463165 | 201176 | 17q21.31 |
| 124 | RAPGEFL1 | Rap guanine nucleotide exchange factor (GEF)-like 1 | Hs.158530 | 51195 | 17q21.2 |
| 125 | HMGCS2 | 3-hydroxy-3-methylglutaryl-Coenzyme A synthase 2 (mitochondrial) | Hs.59889 | 3158 | 1p12 |
| 126 | SYTL2 | synaptotagmin-like 2 | Hs.369520 | 54843 | 11q14 |
| 127 | SLC35D2 | solute carrier family 35, member D2 | Hs.494556 | 11046 | 9q22.33 |
| 128 | HHIP | hedgehog interacting protein | Hs.507991 | 64399 | 4q28-q32 |
| 129 | UNC13B | unc-13 homolog B (C. elegans) | Hs.493791 | 10497 | 9p12-p11 |
| 130 |  | ESTs | Hs.310425 | NA | 9q21.11 |
| 131 | CREB3L1 | cAMP responsive element binding protein 3-like 1 | Hs.405961 | 90993 | 11p11.2 |
| 132 | PTPRH | protein tyrosine phosphatase, receptor type, H | Hs.179770 | 5794 | 19q13.4 |
| 133 | GLYCTK | CG9886-like | Hs.415312 | 132158 | 3p21.31 |
| 134 | HEPH | hephaestin | Hs.31720 | 9843 | Xq11-q12 |
| 135 | CDX2 | caudal type homeo box transcription factor 2 | Hs.174249 | 1045 | 13q12.3 |
| 136 | SPIRE2 | spire homolog 2 (Drosophila) | Hs.461786 | 84501 | 16q24 |
| 137 |  | similar to HSPC323 (LOC284422) | Hs.130714 | 284422 | 19p13.3 |
| 138 | PTPRN2 | protein tyrosine phosphatase, receptor type, N polypeptide 2 | Hs.490789 | 5799 | 7q36 |
| 139 | ORF1-FL49 | putative nuclear protein ORF1-FL49 | Hs.483561 | 84418 | 5q31.3 |
| 140 | FLJ20209 | hypothetical protein FLJ20209 | Hs.528655 | 57245 | 3p21.32 |
| 141 | PLCB3 | phospholipase C, beta 3 (phosphatidylinositol-specific) | Hs.523761 | 5331 | 11q13 |
| 142 | SPIRE2 | spire homolog 2 (Drosophila) | Hs.461786 | 84501 | 16q24 |
| 143 | HOXA13 | homeo box A13 | Hs.414467 | 3209 | 7p15.2 |
| 144 | SPINK4 | serine protease inhibitor, Kazal type 4 | Hs.129778 | 27290 | 9p13.3 |
| 145 | ERN2 | ER to nucleus signalling 2 | NA | 10595 | 16p12.2 |
| 146 | STARD5 | START domain containing 5 | Hs.513075 | 80765 | 15q26 |
| 147 | FXYD3 | FXYD domain containing ion transport regulator 3 | Hs.301350 | 5349 | 19q13.13 |
| 148 | LOC120224 | hypothetical protein BC016153 | Hs.504301 | 120224 | 11q24.3 |
| 149 |  | NA | NA | NA | 1q23.2 |
| 150 |  | hypothetical LOC201484 (LOC201484), mRNA | Hs.51475 | 201484 | 18q11.2 |
| 151 |  | ESTs; strong similarity to hypothetical protein FLJ22593 | Hs.368421 | NA | 16q22.1 |
| 152 | KIAA0711 | KIAA0711 gene product | Hs.5333 | 401444 | 8p23.3 |
| 153 | PLEKHA6 | Pleckstrin homology domain containing, family A member 6 | Hs.253146 | 22874 | 1q32.1 |
| 154 | ADORA2B | adenosine A2b receptor | Hs.167046 | 136 | 17p12-p11.2 |
| 155 |  | ESTs | Hs.436070 | NA | 1p36.33 |
| 156 |  | ESTs; strong similarity to beta-galactosidase (E.coli) | Hs.132441 | NA | 5q23.2 |
| 157 | CKMT1 | creatine kinase, mitochondrial 1 (ubiquitous) | Hs.425633 | 1159 | 15q15 |
| 158 | FLJ22582 | hypothetical protein FLJ22582 | Hs.474822 | 80115 | 22q13.1 |
| 159 | SLC26A6 | solute carrier family 26, member 6 | Hs.436194 | 65010 | 3p21.3 |
| 160 |  | ESTs | Hs.253146 | NA | 1q32.1 |
| 161 | RICS | Rho GTPase-activating protein | Hs.440379 | 9743 | 11q24-q25 |
| 162 | TUBB2 | tubulin, beta, 2 | Hs.433615 | 10383 | 9 |
| 163 | EPB41L4B | erythrocyte membrane protein band 4.1 like 4B | Hs.269180 | 54566 | 9q31-q32 |
| 164 | CTSE | cathepsin E | Hs.1355 | 1510 | 1q31 |
| 165 | DQX1 | DEAQ box polypeptide 1 (RNA-dependent ATPase) | Hs.191705 | 165545 | 2p13.1 |
| 166 | SH3RF2 | SH3 domain containing ring finger 2 | Hs.443728 | 153769 | 5q32 |
| 167 | GNA11 | guanine nucleotide binding protein (G protein), alpha 11 (Gq class) | Hs.435670 | 2767 | 19p13.3 |

**B. Down-regulated genes in MOC compared to other subtypes of ovarian carcinoma.**

| 1 | SOX17 | SRY (sex determining region Y)-box 17 | Hs.98367 | 64321 | 8q11.23 |
| --- | --- | --- | --- | --- | --- |
| 2 | WFDC2 | WAP four-disulfide core domain 2 | Hs.2719 | 10406 | 20q12-q13.2 |
| 3 | SLC34A2 | solute carrier family 34 (sodium phosphate), member 2 | Hs.441716 | 10568 | 4p15.3-p15.1 |
| 4 | TNFAIP2 | tumor necrosis factor, alpha-induced protein 2 | Hs.101382 | 7127 | 14q32 |
| 5 | IGSF4 | immunoglobulin superfamily, member 4 | Hs.156682 | 23705 | 11q23.2 |
| 6 | NA | NA | Hs.43627 | 6666 | NA |
| 7 | ADCY1 | adenylate cyclase 1 (brain) | Hs.259768 | 107 | 7p13-p12 |
| 8 | SOX17 | SRY (sex determining region Y)-box 17 | Hs.98367 | 64321 | 8q11.23 |
| 9 | PAX8 | paired box gene 8 | Hs.308061 | 7849 | 2q12-q14 |
| 10 | KCTD1 | potassium channel tetramerisation domain containing 1 | Hs.433578 | 284252 | 18q12.1 |
| 11 | XPR1 | xenotropic and polytropic retrovirus receptor | Hs.227656 | 9213 | 1q25.1 |
| 12 | UBTD1 | ubiquitin domain containing 1 | Hs.285813 | 80019 | 10q24.2 |
| 13 | HS6ST1 | heparan sulfate 6-O-sulfotransferase 1 | Hs.380792 | 9394 | 2q21 |
| 14 | ESR1 | estrogen receptor 1 | Hs.1657 | 2099 | 6q25.1 |
| 15 | BCL6 | B-cell CLL/lymphoma 6 (zinc finger protein 51) | Hs.155024 | 604 | 3q27 |
| 16 | LOC92312 | cDNA FLJ43493 fis, clone OCBBF3009279 | Hs.529164 | NA | 1q22 |
| 17 | C10orf58 | chromosome 10 open reading frame 58 | Hs.495628 | 84293 | 10q23.1 |
| 18 | CELSR2 | cadherin, EGF LAG seven-pass G-type receptor 2 (flamingo homolog, Drosophila) | Hs.57652 | 1952 | 1p21 |
